# Urine IL-18, NGAL, IL-8 and serum IL-8 are biomarkers of acute kidney injury following liver transplantation

**DOI:** 10.1186/1471-2369-14-17

**Published:** 2013-01-17

**Authors:** Jeffrey C Sirota, Angela Walcher, Sarah Faubel, Alkesh Jani, Kim McFann, Prasad Devarajan, Connie L Davis, Charles L Edelstein

**Affiliations:** 1 Division of Renal Diseases and Hypertension, University of Colorado at Denver Health Sciences Center, Box C281, 12700 East 19th Ave, Aurora, CO 80262, USA; 2 Division Nephrology and Hypertension, Cincinnati Children's Hospital Medical Center, 3333 Burnet Avenue, ML 7022, Cincinnati, OH, USA; 3 Division of Nephrology, Department of Medicine, School of Medicine, University of Washington, Seattle, WA, USA

**Keywords:** Biomarkers, Acute kidney injury, Liver transplantation

## Abstract

**Background:**

AKI is common following liver transplantation and is associated with significant morbidity and mortality. Biomarkers of AKI have not been well established in this setting but are needed to help guide patient care and facilitate development of novel therapeutics.

**Methods:**

Serum creatinine, cystatin C, IL-6, and IL-8 and urine IL-18, NGAL, IL-6, and IL-8 were measured before and within 24 hours after liver transplantation in 40 patients. AKI was defined as a ≥50% sustained increase in creatinine above pre-operative values occurring within 24 hours of transplantation and persisting for at least 24 hours.

**Results:**

Seven patients met criteria for AKI (17.5%), with mean creatinines of 0.81 mg/dL pre-operatively and 1.75 mg/dL post-operatively. While pre-operative biomarker levels in patients with AKI were similar to those in patients without AKI, differences were seen between the groups with regard to median post-operative serum IL-8 (pg/mL) (242.48 vs. 82.37, p = 0.0463) and urine NGAL (ng/mL) (386.86 vs. 24.31, p = 0.0039), IL-6 (pg/mL) (52 vs. 7.29, p=0.0532), IL-8 (pg/mL) (14.3 vs. 0, p = 0.0224), and IL-18 (pg/mL) (883.09 vs. 0, p = 0.0449). The areas under receiver operating characteristic (ROC) curves were 0.749 for urine IL-18, 0.833 for urine NGAL, 0.745 for urine IL-6, 0.682 for serum IL-6, 0.773 for urine IL-8, and 0.742 for serum IL-8. Post-operative cystatin C was not significantly different between AKI and no AKI groups.

**Conclusion:**

Serum IL-8 and urine IL-18, NGAL, IL-6, and IL-8 are elevated in AKI within the first 24 hours following liver transplantation.

## Background

Orthotopic liver transplantation (OLT) is the definitive treatment for end-stage liver disease (ESLD), but the procedure has a significant morbidity and mortality. One of the most significant complications of OLT is AKI, which has been reported in 17 to 95% of OLT patients depending on the definition of AKI [1]. The incidence of AKI was 78% when defined as an increase in serum creatinine of greater than 0.5 mg/dL, 46% when defined as an increase in serum creatinine of greater than 1.0 mg/dL and 14% when defined as an increase in serum creatinine of greater than 50% above 2.0 mg/dL [1]. A variety of etiologies can underlie the renal dysfunction in this setting, including pre-operative hepatorenal physiology, intra-operative vascular clamping that interferes with renal blood flow, peri-operative hypotension and vasopressor requirement, and post-operative use of nephrotoxic medications such as calcineurin inhibitors [2]. These myriad of factors contribute to a high incidence of AKI immediately after liver transplantation, and the development of AKI has been associated with increased length of hospital stay, morbidity, and mortality [1,3-6].

Given the unfavorable outcomes associated with AKI (in the setting of OLT and otherwise), there has been considerable interest in developing therapies to improve renal dysfunction. However, interventional approaches have been limited by the shortcomings of the conventional approach to the diagnosis of AKI. Measurement of serum creatinine remains the commonest diagnostic tool for assessment of renal function, but this long-used marker suffers from its insensitivity to small changes in renal function, its lack of specificity for renal function given the influence exerted by a variety of non-renal factors (such as muscle mass and diet), and its long lag-time in response respond to kidney injury. These problems can be particularly pronounced in the setting of ESLD, where creatinine has been shown to be an especially unreliable marker of renal function [7]. The inadequacies of creatinine-based estimates of renal function have probably limited the success of AKI therapeutic trials and the opportunity to change the therapeutic strategy accordingly (e.g., delaying the initiation of nephrotoxic immunosuppression after transplant in patients with AKI). A new class of molecular biomarkers has shown significant promise in other clinical settings. These biomarkers include serum cystatin C, urine IL-6, serum IL-6, urine IL-18, and urinary neutrophil gelatinase-associated lipocalin (NGAL). These molecules have been shown to be early detectors of AKI in a variety of clinical settings such as critical illness, [8-10] after cardiopulmonary bypass surgery, [11,12] in heart failure, [13] and with contrast-induced kidney injury[14].

However, the role of biomarkers for detection of AKI in patients undergoing OLT has been explored in only a few prior studies that were limited to urinary and serum NGAL levels [15-18]. Urinary NGAL and L-FABP have recently been described as biomarkers of early AKI after liver transplantation [19]. Besides NGAL and L-FABP, there have been no reports of using other established AKI biomarkers in OLT patients, in whom the levels of these molecules could be altered by factors unrelated to kidney function. Therefore, in this study, we examined a wider panel of biomarkers of AKI in patients undergoing OLT, with the hypothesis that post-operative biomarkers would be higher in patients who develop AKI than in those who do not.

## Methods

### Patients

All patients aged 18 to 90 years old who underwent a first-time OLT at the University of Colorado (N=11) and University of Washington (N=29) were eligible for the study. Patients were excluded if they were pregnant, incarcerated, unable to provide consent, received dialysis before or during OLT, or received a kidney transplant simultaneously with their liver, had pre-renal AKI (a more than 50% rise in serum creatinine that was not sustained for 24 hours). All the patients received either tacrolimus or cyclosporine as immunosuppressive therapy. All study enrollment procedures and subsequent data collection and acquisition was approved by the Colorado Multiple Institutional Review Board. All eligible patients who were consented according to proper procedures in a consecutive fashion over an 18 month period were enrolled at the 2 hospitals.

### Sample collection and processing

Pre-operative blood and urine samples (four and ten milliliters, respectively) were collected from each patient within 24 hours of OLT. Additional samples of the same volumes were collected within 24 hours after OLT (measured from the time of surgical incision, blood samples were collected an average of 21 hours and 41 minutes later and urine samples were collected an average of 21 hours and 38 minutes later. Average time point between surgical incision and time to first blood sample was 21 hours and 21 minutes in the AKI group. Collected urine was centrifuged at 5000G for two minutes, and the supernatant liquid was stored at −80°C. Blood samples were centrifuged at 3500 RPM for ten minutes, and serum was stored at −80°C.

Biomarkers were measured on single, not pooled samples. Serum cystatin C levels were measured using an immunonephelometric method (Dade Behring, Marburg, Germany), and a standard calibration formula was used to estimate glomerular filtration rate from the result [20]. Urine IL-18 levels were measured with a specific enzyme-linked immunosorbent assay (ELISA) kit (Medical and Biological Laboratories, Nagoya, Japan) that specifically detects the mature form of IL-18 as previously described [10]. All measurements were made in a blinded fashion. The inter- and intra-assay coefficient variations were 5–10%, corresponding to that reported by the kit manufacturer. Urine NGAL levels were measured using a commercially available ELISA (Bioporto, Gentofte, Denmark) as published previously [11]. Briefly, microtiter plates pre-coated with a mouse monoclonal antibody raised against human NGAL (HYB211–05, AntibodyShop, Gentofte, Denmark) were blocked with buffer containing 1% BSA, coated with 100 μL of samples (urine) or standards (NGAL concentrations ranging from 1–1000 ng/mL), and incubated with a biotinylated monoclonal antibody against human NGAL (HYB211–01B, AntibodyShop) followed by avidin-conjugated HRP (Dako, Carpinteria, CA, USA). TMB substrate (BD Biosciences, San Jose, CA, USA) was added for color development, which was read after 30 min at 450 nm with a microplate reader (Benchmark Plus, BioRad, Hercules, CA, USA). The intra- and inter-assay coefficients of variability (CVs) were 5.6% and 6.4%, respectively. Serum and urine IL-6 and IL-8 levels were measured using an ELISA kit (R&D Systems, Minneapolis, MN). Briefly, the IL-6 and IL-8 assays employ the quantitative sandwich enzyme immunoassay technique. A monoclonal antibody specific for IL-6 or IL-8 has been pre-coated onto a microplate. Standards and samples are pipetted into the wells and any IL-6 or IL-8 present is bound by the immobilized antibody. After washing away any unbound substances, an enzyme-linked polyclonal antibody specific for IL-6 or IL-8 is added to the wells. Following a wash to remove any unbound antibody-enzyme reagent, a substrate solution is added to the wells and color develops in proportion to the amount of IL-6 bound in the initial step. The color development is stopped and the intensity of the color is measured.

Serum creatinine levels were measured daily by the hospital’s central laboratory as part of routine peri-operative care, and these values were recorded. A combination of retrospective and prospective chart reviews was performed to collect demographic and pertinent clinical data. AKI was defined as an increase in serum creatinine by 50% or greater compared to pre-operative values that occurred within 24 hours of OLT and that was sustained for at least 24 hours. Patients who developed a 50% rise in serum creatinine that was not sustained for 24 hours were removed from analysis as their kidney injury was thought to be pre-renal in nature. The definition of AKI was based on the Risk, Injury, Failure; Loss, End-Stage Renal Disease (RIFLE) classification and Acute Kidney Injury Network (AKIN) classification where Risk or AKIN stage 1 represents an increase of serum creatinine to 1.5 times baseline [21]. In a separate analysis, biomarkers were compared in patients with or without AKI as defined by a >50% increase in serum cystatin C level at 24 hours.

### Statistics

Demographic data was compared between patients who developed AKI and those who did not using either the Fisher’s exact test or the unpaired *t* test. Two-tailed p-values are presented. Mean and standard deviation calculations were performed for the measured levels of serum creatinine and serum cystatin C. Mean pre-operative values were compared to mean post-operative values among patients who developed AKI and separately among patients who did not develop AKI using two-tailed paired *t* tests. At each of the two timepoints (pre-operative and post-operative), mean creatinine and cystatin C values among patients who developed AKI were compared to those in patients who did not develop AKI using two-tailed unpaired *t* tests. For serum and urinary biomarkers, medians and interquartile ranges were calculated, and median values of the AKI group were compared to those of the no AKI group using the Mann–Whitney test with two-tailed p-values. We defined statistical significance as a p-value of <0.05. InStat software was used for statistical analysis.

## Results

Table 1 shows the demographic information and clinical characteristics of patients who underwent liver transplantation in this study. Seven patients met the criteria for AKI (“AKI group”) and 33 did not (“no AKI group”). The mean age of patients who developed AKI was 58.9 years, compared to 55.6 years in patients who did not develop AKI. The AKI group was 55.6% male and 88.9% Caucasian, compared to 64.7% male and 82.4% Caucasian in the group without AKI. The two patient groups had statistically similar prevalences of hypertension and diabetes mellitus (44.4% vs. 23.5% for hypertension in the AKI vs. no AKI groups, respectively; 22.2% vs. 26.5% for diabetes mellitus in the AKI vs. no AKI groups, respectively), and they had similar baseline serum creatinine values (0.89 vs. 1.07 mg/dL), which were determined from mean serum creatinine values obtained within three months prior to transplant. Additionally, there was no statistically significant difference in the two groups with regard to average duration of end-stage liver disease prior to transplant (75.4 vs. 99.2 months), pre-operative Model for End Stage Liver Disease (MELD) scores (16.3 vs. 18.6), or prevalences of viral or alcoholic etiologies for their cirrhosis. Duration of surgery was longer in the AKI group compared to the no AKI group (Table 1). There was no difference in AST, ALT, alkaline phosphatase, albumin or bilirubin pre-operatively between AKI and non AKI patients. AST (U/L) was 84 in the AKI group and 95 in the non-AKI group (P=NS). ALT (U/L) was 43 in the AKI group and 68 in the non-AKI group (P=NS). Alkaline phosphatase (U/L) was 130 in the AKI group and 135 in the non-AKI group (P=NS). Serum albumin (g/dL) was 3.15 in the AKI group and 3.08 in the non-AKI group (P=NS). Bilirubin (mg/dL) was 2.4 in the AKI group and 3.6 in the non-AKI group (P=NS). X.

**Table 1:** Demographic information in patients undergoing liver transplantation

	**AKI**	**no AKI**	**p-value**
	**(N=7)**	**(N=33)**	
age (years)*	58.9	55.6	0.26 ^a^
	(3.6)	(7.2)	
% male	57.1	69.7	0.66 ^b^
% Caucasian	85.7	93.5	0.47 ^b^
baseline serum creatinine (mg/dL)*	0.89	1.07	0.21 ^a^
	(0.30)	(0.30)	
duration of ESLD (months)*	75.4 (37.8)	99.2 (79.1)	0.45 ^a^
etiology of ESLD			
% with a viral component	85.7	51.5	0.21 ^b^
% with an alcoholic component	14.3	33.3	0.99 ^b^
pre-operative MELD score*	16.3 (8.4)	18.6 (5.4)	0.36 ^a^
history of hypertension (%)	57.1	24.2	0.17 ^b^
history of diabetes (%)	14.3	30.3	0.65 ^b^
Duration of surgery (hours)	6.0	4.6	<0.01

*data presented as mean (SD).

^a^ comparison made using unpaired *t* test.

^b^ comparison made using Fisher’s exact test.

Table 2 shows mean creatinine values for the AKI and no AKI groups. The mean serum creatinine measured within 24 hours prior to liver transplant was 0.81 mg/dL in patients who subsequently developed AKI, as manifested by a mean post-operative serum creatinine of 1.75 mg/dL. This increase was statistically significant (p = 0.0002). In contrast, patients in the no AKI group had a mean pre-operative serum creatinine value of 1.13 mg/dL, and post-operatively their mean serum creatinine was 1.21 mg/dL. This change in serum creatinine was not statistically significant (p = 0.0809). As expected, the AKI group’s mean post-operative creatinine was significantly higher than that of the group without AKI (1.75 vs. 1.21 mg/dL, p = 0.0005). A statistically significant difference between the AKI and no AKI group was also seen with regard to mean pre-operative creatinine as well (0.81 vs. 1.13 mg/dL, p = 0.0131). Median values for pre-operative creatinine were 0.80 mg/dL for the AKI group and 1.10 mg/dL for the no AKI group; median post-operative values were 1.8 mg/dL and 1.2 mg/dL, respectively.

**Table 2:** Creatinine and cystatin C measurements (means)

	**AKI**	**no AKI**	p-value*
	**(N=7)**	**(N=33)**	
**Mean serum creatinine in mg/dL (SD)**			
pre-operative	0.81 (0.17)	1.13 (0.32)	0.0131
post-operative	1.75 (0.27)	1.21 (0.35)	0.0005
**Mean serum cystatin C in mg/L (SD)**			
pre-operative	0.94 (0.30)	1.16 (0.39)	0.1674
post-operative	1.33 (0.51)	1.19 (0.42)	0.4236

*two-tailed p-values based on unpaired *t* tests.

To determine whether low albumin or systemic inflammation contributed to the lower pre-operative creatinine in the AKI group, serum albumin and serum IL-6 was compared between the AKI and no AKI groups. There was no difference in serum albumin or serum IL-6 pre-op between the AKI and no AKI groups suggesting that malnutrition or systemic inflammation is not the cause of the lower serum creatinine in the AKI group: Serum albumin (g/dL) pre-op was 3.15 in the AKI group and 3.08 in the non-AKI group (P=NS). Serum IL-6 (pg/mL) pre-op was 20.2 in the AKI group and 15.0 in the non-AKI group (P=NS).

Table 2 also shows the mean serum cystatin C values for the two groups before and after liver transplantation. Patients who developed AKI had a mean pre-operative cystatin C value of 0.94 mg/L, compared to the no AKI group’s mean value of 1.16 mg/L (p = 0.1674). These two groups did not have a statistically significant difference in their post-operative mean cystatin C values (1.33 for the AKI group vs. 1.19 mg/dL for the no AKI group, p = 0.4236). While the AKI group did have a substantial increase in serum cystatin C value (0.94 mg/L pre-operatively, 1.33 mg/L post-operatively), the increase failed to reach statistical significance (p = 0.0812). The change in serum cystatin C level amongst the no AKI patient group was small and not statistically significant (1.16 mg/L pre-operatively, 1.19 mg/L post-operatively; p = 0.6325). Median values for pre-operative cystatin C were 0.94 mg/L for the AKI group and 1.19 mg/L for the no AKI group; median post-operative values were 1.36 mg/L and 1.16 mg/L, respectively.

Table 3 shows median pre-operative and post-operative biomarker levels in patients who developed AKI and those who did not. On average, urine and serum biomarkers were collected less than 22 hours after surgical incision (1298 minutes for urine samples, 1301 minutes for blood). All pre-operative biomarker levels were statistically similar when comparing AKI and no AKI groups (p-values ranging from 0.1577 to 0.6693). When comparing post-operative biomarker levels in patients with AKI to those without AKI, statistically significant differences were seen in urine IL-18 (pg/mL) (883.09 vs. 0, p = 0.0449), urine NGAL (ng/mL) (386.86 vs. 24.31, p = 0.0039), urine IL-8 (pg/mL) (14.3 vs. 0, p=0.0224) and serum IL-8 (pg/mL) (242.48 vs. 82.37, p = 0.0463). A trend towards significant difference was seen for urine IL-6 (pg/mL) (52.00 vs. 7.29 in the AKI and no AKI groups, respectively, p = 0.0532). The difference between serum IL-6 values in the two groups was different (44.76 vs. 18.75), but the p-value did not reach statistical significance (p = 0.1025).

**Table 3:** Median biomarker levels [25-75% IQR], (range) when AKI is defined as a >50% increase in serum creatinine

	**AKI**	**no AKI**	**p-value**
**Urine IL-18 (pg/mL)**			
pre-op	0	0	0.4274
	[0–18.57]	[0–0]	
	(0–1369)	(0–32.16)	
post-op	884.09	0	0.0449
	[0–5024.4]	[0–42.73]	
	(0–5024.4)	(0–4915.6)	
**Urine NGAL (ng/mL)**			
pre-op	20.27	6.52	0.1848
	[4.70-272.12]	[3.41-10.76]	
	(0.97-8672.2)	(0.05-56.82)	
post-op	386.86	24.31	0.0039
	[71.38-1611.48]	[9.97-45.20]	
	(41.23-1824.6)	(1.69-5522.1)	
**Urine IL-6 (pg/mL)**			
pre-op	6.82	6.34	0.1577
	[4.28-50.11]	[1.59-9.36]	
	(1.90-376.58)	(0–40.59)	
post-op	52.00	7.29	0.0532
	[5.55-77.22]	[3.81-13.16]	
	(20.61-84.04)	(0.48-130.34)	
**Serum IL-6 (pg/mL)**			
pre-op	14.21	10.28	0.6693
	[5.75-24.19]	[6.05-17.54]	
	(3.33-67.14)	(1.21-60.18)	
post-op	44.76	18.75	0.1025
	[17.54-133.67]	[9.38-38.71]	
	(13–3020.6)	(4.23-171.77)	
**Urine IL-8 (pg/mL)**			
pre-op	0	0	0.1881
	[0–131.70]	[0–0]	
	(0–200.1)	(0–143.2)	
post-op	14.30	0	0.0224
	[0–143.00]	[0–0]	
	(0–163.8)	(0–238.8)	
**Serum IL-8 (pg/mL)**			
pre-op	12.36	6.53	0.6175
	[3.50-30.51]	[0–36.35]	
	(0–114.78)	(0–786.23)	
post-op	242.48	82.37	0.0463
	[97.28-341.00]	[20.14-133.58]	
	(41.53-1267.5)	(0–598.99)	

The absolute change in serum creatinine (mg/dl) from pre-operative to 24, 48, 72, 96 and 120 hrs post-operative in AKI patients is shown in Table 4. In 3 of the 7 AKI patients the serum creatinine continued to rise at 48 hours post-operative. In 4 of the 7 AKI patients, the serum creatinine remained increased by 0.3 or more at 120 hours post-operative.

**Table 4:** Absolute change in serum creatinine (mg/dl) from pre-operative to 24, 48, 72, 96 and 120 hrs post-operative in AKI patients

	**24 hr**	**48 hr**	**72 hr**	**96 hr**	**120 hr**
Patient 1	0.9	0.5	0.2	0.2	0.2
Patient 2	0.9	1.2	0.9	0.3	0.8
Patient 3	0.6	0.5	0.5	0.7	0.3
Patient 4	1.1	0.4	0.2	0.1	0.1
Patient 5	0.6	0.4	0.2	−0.1	0.1
Patient 6	1.5	3.4	3.7	2.6	1.5
Patient 7	1.1	2.0	1.2	0.4	0.4

**Figure 1:**
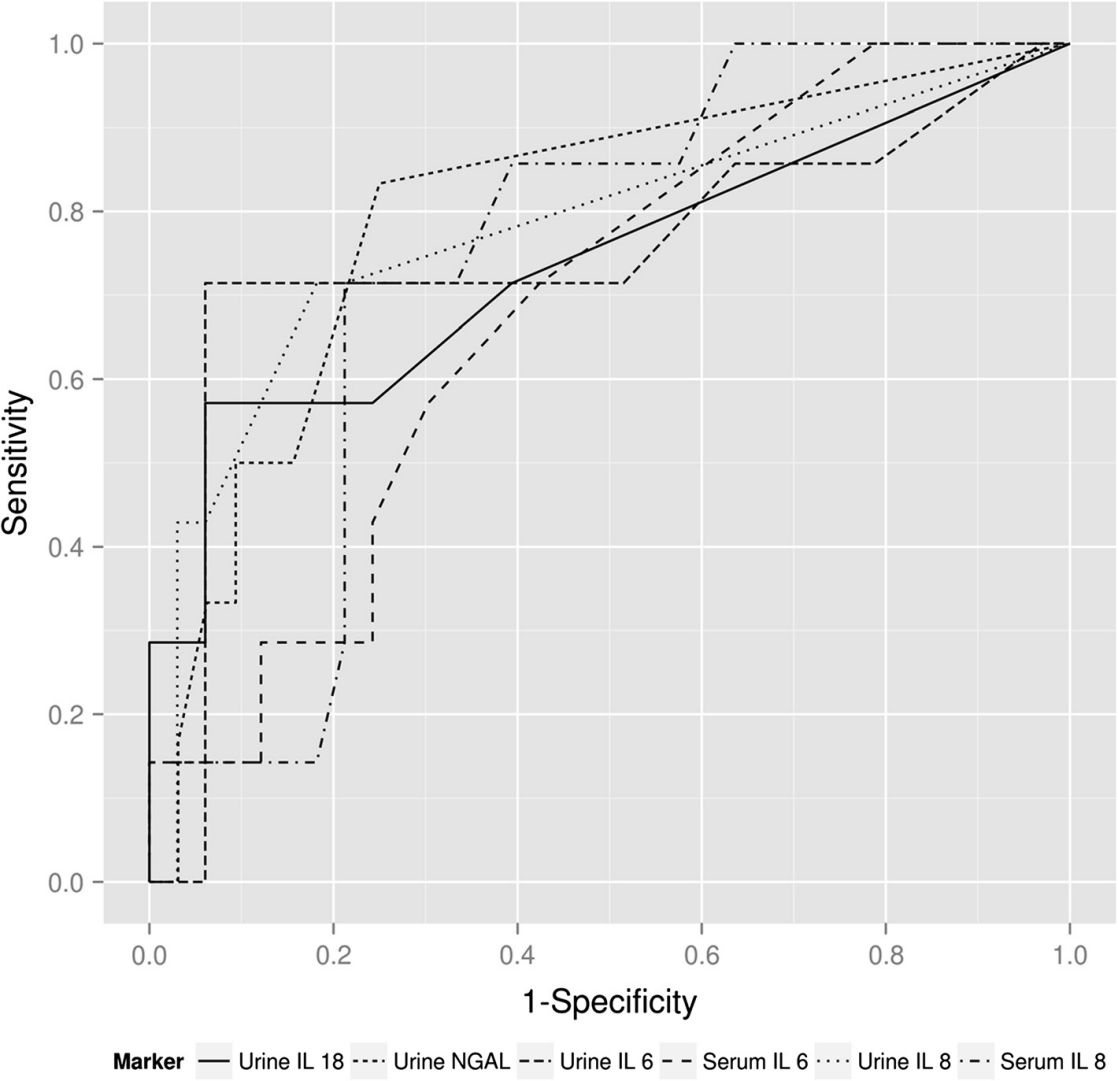
**Receiver operator characteristic (ROC) curves for post-operative biomarker levels.** P-values for these analyses are the following: 0.0384 for urine IL-18, 0.3671 for urine NGAL, 0.0296 for urine IL-6, 0.6682 for serum IL-6, 0.0949 for urine IL-8, 0.0803 for serum IL −8.

Figure 1 shows receiver operating characteristic (ROC) curves for the post-operative biomarker levels. The area under the curve (AUC) for each of the ROC curves is the following: 0.749 for urine IL-18, 0.833 for urine NGAL, 0.745 for urine IL-6, 0.682 for serum IL-6, 0.773 for urine IL-8, and 0.742 for serum IL-8.

Finally, we repeated the biomarker analysis using a different definition of AKI. Seven patients met the criteria of AKI as defined by a >50% increase in serum cystatin C level at 24 hours. These seven patients had no statistical differences from the non-AKI patients in terms of age, sex, race, pre-operative MELD score, or baseline serum creatinine level, or pre-operative serum creatinine level. As shown in Table 5, all pre-operative biomarker levels were found to be statistically similar between AKI and non-AKI groups, but post-operative urine NGAL, IL-6, and IL-8 levels in the AKI group were all found to be higher (with statistical significance) when compared to the non-AKI group.

**Table 5:** Median biomarker levels (range) when AKI is defined as a >50% increase in cystatin C

	**AKI**	**no AKI**	**p-value**
**Urine IL-18 (pg/mL)**			
pre-op	0	0	0.932
	(0–18.57)	(0–1369.00)	
post-op	85.54	1.73	0.354
	(0–5024.4)	(0–5024.4)	
**Urine NGAL (ng/mL)**			
pre-op	7.41	7.24	0.712
	(0.97-32.75)	(0.05-8672.2)	
post-op	386.86	24.98	0.0144
	(33.76-5522.1)	(1.69-1655.2)	
**Urine IL-6 (pg/mL)**			
pre-op	19.03	5.95	0.2236
	(0–50.11)	(0–376.58)	
post-op	33.14	7.37	0.0171
	(5.55-130.34)	(0.48-105.13)	
**Serum IL-6 (pg/mL)**			
pre-op	16.03	8.92	0.8344
	(3.33-24.19)	(1.21-67.14)	
post-op	17.54	20.26	0.6637
	(7.26-3020.6)	(4.23-171.77)	
**Urine IL-8 (pg/mL)**			
pre-op	0	0	0.6468
	(0–131.70)	(0–200.10)	
post-op	14.30	0	0.0184
	(0–238.8)	(0–106.30)	
**Serum IL-8 (pg/mL)**			
pre-op	9.12	3.26	>0.9999
	(0–107.00)	(0–786.23)	
post-op	97.28	83.99	0.3318
	(33.10-1267.50)	(0–598.99)	

## Discussion

In this study, we sought to evaluate the performance of a variety of biomarkers of AKI in the setting of OLT when examined within 24 hours of surgery. We used serum creatinine in our definition of AKI because of its prominence in standard AKI definitions and because data on the superiority of cystatin C in the setting of OLT is conflicting. Some studies have shown that cystatin C may be a better assay for GFR estimation in the setting of cirrhosis [22] and a 2006 study showed that serum cystatin C correlated better with iohexol-based GFR estimates than serum creatinine did [23]. However, a subsequent study in 2009 did not demonstrate improved diagnostic accuracy of cystatin C over serum creatinine in the setting of liver transplantation [24]. Serum cystatin C has been shown to be superior to creatinine as a marker of kidney function in chronic kidney disease [25]. Therefore, we chose to use serum creatinine in our AKI definition despite its limitations.

As established by our definition, post-operative serum creatinine levels were higher in the AKI group than in the non-AKI group. Interestingly, however, we found that pre-operative serum creatinine levels were significantly lower in the AKI group than in the non-AKI group. A similar pattern was seen with respect to cystatin C (although the difference in pre-operative cystatin C levels failed to achieve statistical significance). While these differences were not expected, they supported the notion that AKI patients did not harbor any manifest renal dysfunction prior to transplantation, and the similarities seen in the pre-operative biomarker levels between the groups further supports this hypothesis.

Using serum creatinine elevations to define AKI, we found statistically significant elevations in post-operative urine IL-18, urine NGAL, urine IL-8, and serum IL-8 in patients with AKI compared to those without AKI, and both serum and urine IL-6 levels were higher in AKI patients despite failing to achieve statistical significance. ROC curve analysis showed that urine NGAL had the best diagnostic performance and that the IL-6 measures performed the worst amongst those we tested. We also examined the biomarkers using an alternative, cystatin C-based definition of AKI. With AKI defined as a >50% increase in serum cystatin C levels at 24 hours, we found that post-operative urine NGAL, IL-6, and IL-8 levels were all significantly elevated above non-AKI controls. We did not normalize urinary biomarker concentrations to contemporaneous urine creatinine concentration because urinary creatinine excretion in the setting of AKI is dynamic and its inclusion in the assessment of urinary biomarkers has been challenged [26].

While a few previous reports have documented elevations in NGAL levels in post-liver transplant patients who develop AKI, the utility of other biomarkers that have been proven indicators of AKI has not yet been established in the setting of OLT. In 2009, Niemann et al. evaluated plasma NGAL levels in 59 patients undergoing OLT and found that elevations in NGAL levels measured two hours after reperfusion was predictive of AKI [17]. Among those patients with pre-operative serum creatinine levels of <1.5 mg/dL, plasma NGAL level at two hours post-perfusion was associated with the subsequent development of AKI. The following year, Portal et al. evaluated both urinary and serum NGAL levels in 95 patients undergoing OLT and found that post-operative serum NGAL (but not urinary NGAL) was a predictor of severe AKI in multiple logistic regression analysis [18]. In 2011, Wagener et al. examined urinary NGAL/creatinine ratios in 92 patients undergoing OLT [15]. Elevations in urinary NGAL/creatinine ratios were detected three hours after reperfusion and were more pronounced in patients who developed AKI. Urinary NGAL/creatinine ratios were evaluated again in a recent study by Jeong et al. [16]. Elevated urinary NGAL/creatinine ratios were seen two hours after reperfusion in 11 patients who developed AKI after living-related liver transplantation, and peak elevation preceded the rise in serum creatinine in these patients by 19 hours.

Despite this data on NGAL’s role as a predictor of AKI, other biomarkers have not been evaluated in the setting of OLT. The need for studying other biomarkers in AKI is underscored by a closer examination of the previous studies on urinary NGAL that reveals some limitations of its diagnostic ability. When ROC curves were constructed in these prior studies, the AUC’s did not indicate superior diagnostic capabilities (AUC’s for urinary NGAL ranged from 0.693 to 0.800). Thus, urinary NGAL may be an imperfect tool in the early detection of AKI, and defining the diagnostic characteristics for other biomarkers may prove useful in designing future studies to determine whether multiple biomarkers are early predictors of AKI [27].

Our goal was to expand the current panel of biomarkers that has been studied in the setting of OLT from NGAL alone to include urine IL-18, serum and urine IL-6, and serum and urine IL-8. These various biomarkers have been established in other settings. IL-18, a proinflammatory cytokine, has been identified as a mediator of ischemic injury in the kidney and has been demonstrated to be a reliable marker of and contributor to AKI through animal studies [28-30]. Further studies have demonstrated its diagnostic utility in patients with AKI in the setting of critical illness [10,31] following cardiopulmonary bypass surgery [12,32-34] and after toxin exposure [14,35] IL-6 is another pro-inflammatory cytokine that has been shown to be elevated in animal models of AKI [36] and in humans with AKI after cardiac surgery [37,38] or with severe sepsis [39]. IL-8 is an endothelial-derived chemokine involved in recruiting neutrophils to the site of injury and stimulating their response. IL-8 levels have been shown to be elevated in the setting of renal allograft dysfunction [40] and in AKI associated with cardiopulmonary bypass surgery [41].

It has been reported that worst pre-orthotopic liver transplant hepatic function is associated with an increased incidence of post- orthotopic liver transplant AKI [42,43]. In the present study, there was no difference in MELD score between AKI and non AKI patients. Also, there was no difference in pre-operative AST, ALT, alkaline phosphatase, albumin or bilirubin between AKI and non AKI patients.

Our study has limitations. First, the overall sample size was small and the incidence of post-operative AKI was significantly lower than that reported in the literature, resulting in a small pool of patients for analysis. This shortcoming may have diminished our statistical power and precluded a more robust calculation of our biomarkers’ predictive abilities. Second, it is likely that some of these biomarkers would have been elevated earlier than 24 hours post-operatively. Thus biomarkers that rise quickly in response to AKI may have already peaked and began normalizing by the time we measured them at 24 hours post-operatively. The degree of elevation may have underestimated the peak degree of elevation and thereby diminished our diagnostic power. Additionally, we may have missed transient elevations that had fully resolved by the time we collected our samples. In the present study, biomarkers were measured at the same time as serum creatinine and were elevated at the same time as serum creatinine in AKI patients. Our previous studies in AKI patients post cardiac surgery demonstrate that urine IL-18 and NGAL peak at 6 hrs post op [34]. Thus it is possible that urine IL-18 and NGAL may peak earlier than serum creatinine and serve as an early biomarker of AKI post liver transplant. Future studies will determine whether these biomarkers increase before serum creatinine in liver transplant patients.

## Conclusion

In conclusion, the present study establishes the utility of measuring a wider selection of cytokines and chemokines (urine IL-18, urine NGAL, urine IL-8, serum IL-8 and urine IL-6) as biomarkers of AKI post liver transplantation. The present study should prompt further research into using these biomarkers with urine NGAL to detect AKI prior to randomization to therapeutic strategies in clinical studies. Given the prevalence of AKI associated with OLT and its associated morbidity and mortality, therapeutic studies based on the diagnosis of AKI using a panel of biomarkers including kidney injury molecule-1 (KIM-1), may be possible in the future. The development of novel therapies for AKI may lead to better outcomes.

## Competing interests

There are no conflicts of interest to declare.

## Authors’ contribution

JS and AW randomized patients, collected samples, set up database, analyzed data, wrote manuscript. SF and AJ analyzed data, wrote paper. KM did statistical analysis. PD measured NGAL and data analysis. CD randomized patients, collected samples, advised on data analysis. CE data analysis, wrote paper. All authors read and approved the final manuscript.

## Pre-publication history

The pre-publication history for this paper can be accessed here:

http://www.biomedcentral.com/1471-2369/14/17/prepub
